# 5-Amino-1,3,4-thia­diazol-2(3*H*)-one

**DOI:** 10.1107/S1600536812012433

**Published:** 2012-03-28

**Authors:** Sung Kwon Kang, Nam Sook Cho, Siyoung Jang

**Affiliations:** a Department of Chemistry, Chungnam National University, Daejeon 305-764, Republic of Korea

## Abstract

The asymmetric unit of the title compound, C_2_H_3_N_3_OS, contains three independent mol­ecules which are essentially planar, with r.m.s. deviations of 0.011 (2)–0.027 (2) Å from the mean plane defined by the seven non-H atoms. In the crystal, N—H⋯N and N—H⋯O hydrogen bonds link the mol­ecules into a sheet parallel to the (111) plane.

## Related literature

For the structures and reactivity of thia­diazole derivatives, see: Parkanyi *et al.* (1989 ▶); Cho, Cho *et al.* (1996 ▶); Cho, Ra *et al.* (1996 ▶). For the biological activity of thia­diazole derivatives, see: Castro *et al.* (2008 ▶); Ra, Cho & Cho (1998 ▶); Ra, Cho, Moon & Kang (1998 ▶).
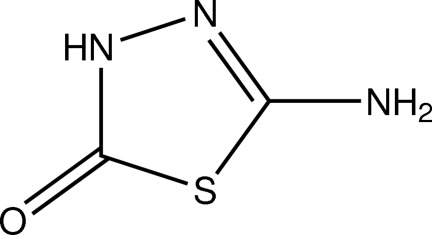


## Experimental

### 

#### Crystal data

C_2_H_3_N_3_OS
*M* *_r_* = 117.13
Triclinic, 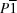

*a* = 7.2860 (2) Å
*b* = 10.2982 (3) Å
*c* = 10.7727 (3) Å
α = 63.721 (3)°
β = 73.122 (2)°
γ = 76.737 (2)°
*V* = 688.74 (3) Å^3^
*Z* = 6
Mo *K*α radiation
μ = 0.57 mm^−1^
*T* = 296 K
0.15 × 0.1 × 0.05 mm

#### Data collection

Bruker SMART CCD area-detector diffractometer
Absorption correction: multi-scan (*SADABS*; Bruker, 2002 ▶) *T*_min_ = 0.93, *T*_max_ = 0.97
23857 measured reflections
3433 independent reflections
2526 reflections with *I* > 2σ(*I*)
*R*_int_ = 0.055

#### Refinement

*R*[*F* ^2^ > 2σ(*F* ^2^)] = 0.031
*wR*(*F* ^2^) = 0.080
*S* = 0.94
3433 reflections
226 parameters
All H-atom parameters refined
Δρ_max_ = 0.30 e Å^−3^
Δρ_min_ = −0.26 e Å^−3^

### 

Data collection: *SMART* (Bruker, 2002 ▶); cell refinement: *SAINT* (Bruker, 2002 ▶); data reduction: *SAINT*; program(s) used to solve structure: *SHELXS97* (Sheldrick, 2008 ▶); program(s) used to refine structure: *SHELXL97* (Sheldrick, 2008 ▶); molecular graphics: *ORTEP-3* (Farrugia, 1997 ▶); software used to prepare material for publication: *WinGX* (Farrugia, 1999 ▶).

## Supplementary Material

Crystal structure: contains datablock(s) global, I. DOI: 10.1107/S1600536812012433/is5096sup1.cif

Structure factors: contains datablock(s) I. DOI: 10.1107/S1600536812012433/is5096Isup2.hkl

Supplementary material file. DOI: 10.1107/S1600536812012433/is5096Isup3.cml

Additional supplementary materials:  crystallographic information; 3D view; checkCIF report

## Figures and Tables

**Table 1:** Hydrogen-bond geometry (Å, °)

*D*—H⋯*A*	*D*—H	H⋯*A*	*D*⋯*A*	*D*—H⋯*A*
N3—H3⋯N18	0.854 (19)	2.004 (19)	2.8516 (19)	171.9 (18)
N7—H7*A*⋯O20^i^	0.87 (2)	2.07 (2)	2.907 (2)	160 (2)
N10—H10⋯N4	0.98 (2)	1.88 (2)	2.8558 (19)	175.7 (18)
N14—H14*A*⋯O6^ii^	0.83 (2)	2.10 (2)	2.897 (2)	162 (2)
N17—H17⋯N11	0.88 (2)	1.97 (2)	2.8424 (18)	179 (4)
N21—H21*A*⋯O13^iii^	0.80 (3)	2.10 (3)	2.878 (2)	163 (2)

Symmetry codes: (i) 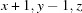; (ii) 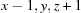; (iii) 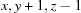.

**Fig. 1.**
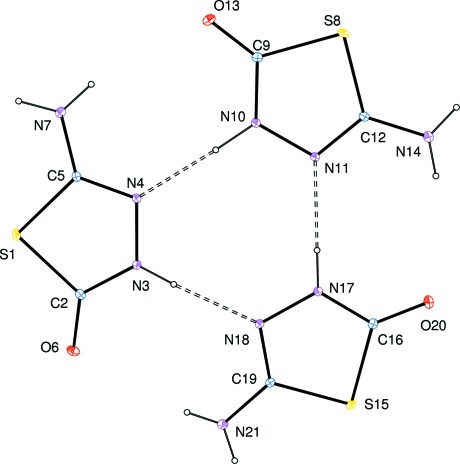
Molecular structure of the title compound, showing the atom-numbering scheme and 30% probability ellipsoids. Intermolecular N—H···N hydrogen bonds are indicated by dashed lines.

**Fig. 2.**
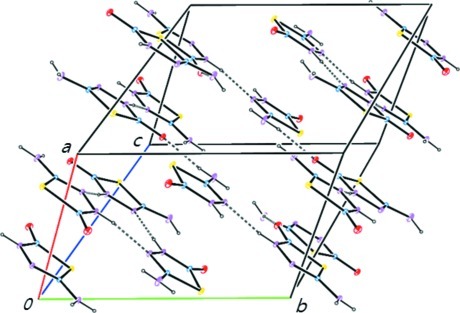
Part of the crystal structure of the title compound, showing molecules linked by intermolecular N—H···N and N—H···O hydrogen bonds (dashed lines).

## supplementary crystallographic information

### Comment

5-Amino-2*H*-1,2,4-thiadiazolin-3-one heterocycle is an analog of cytosine (Parkanyi *et al.*, 1989). Derivatives of 5-amino-2*H*-1,2,4-thiadiazolin-3-one have recently attracted attention on the antibacterial activity, potential carcinogenicity, and kinase inhibitor activity (Castro *et al.*, 2008; Cho, Ra *et al.*, 1996; Ra, Cho, Moon & Kang, 1998). 5-Amino-3*H*-1,3,4-thiadiazolin-2-one is an isomer of 5-amino-2*H*-1,2,4-thiadiazolin-3-one, which has become an attractive moiety due to potential biological activities (Cho, Cho, Ra, Moon *et al.*, 1996; Ra, Cho & Cho 1998).

In (I), three independent but similar molecules, which are linked by the intermolecular N—H···N hydrogen bonds (Fig. 1), comprise the asymmetric unit. The 1,3,4-thiadiazolin-2-one units are almost planar with r.m.s. deviations of 0.011 (2)–0.027 (2) Å from the corresponding least-squares plane defined by the seven constituent atoms. The bond distance of N4—C5 [1.291 (2) Å; N11—C12, 1.287 (2) Å; N18—C19, 1.282 (2) Å] is shorter than that of C2—N3 [1.333 (2) Å; C9—N10, 1.336 (2) Å; C16—N17, 1.327 Å], which is consistent with double bond character. The crystal structure is stabilized by the intermolecular N—H···N and N—H···O hydrogen bonds, which link the molecules into a two-dimensional sheet parallel to the (111) plane (Table 1 and Fig. 2).

### Experimental

Synthesis of 5-amino-2-ethoxy-1,3,4-thiadiazole: Ethyl thiocarbazate (4.8 g, 0.04 mol) was dissolved in 24 ml of 2 N NaOH at 10 °C. Cyanogen bromide (4.2 g, 0.04 mol) dissolved in 20 ml of ethanol was added to the above solution keeping the temperature below 10 °C during 45 minutes. The solid product (4.1 g, 71%) was collected by filtration. To obtain the analytical sample the product was recrystallized from ethanol. m.p. 200–202 °C; IR (KBr, cm^-1^) 3300 (NH), 3150 (NH), 3000 (CH), 2950 (CH), 1620 (C=O), 1580 (C=N); ^1^H NMR (DMSO-d_6_, p.p.m.) 6.65 (2*H*, b, NH_2_), 4.25 (2*H*, q, CH_2_), 1.29 (3*H*, t, CH_3_); ^13^C NMR (DMSO-d_6_, p.p.m.) 164.85 (C=N), 162.18 (C—O), 67.48 (CH_2_), 14.35 (CH_3_); Anal. Calcd. For C_4_H_7_N_3_OS: C 33.09, H 4.86, N 28.94. Found: C 33.71, H 4.94, N 28.50.

Synthesis of title compound: 5-Amino-2-ethoxy-1,3,4-thiadiazole (5 g, 34.5 mmol) was dissolved in 50 ml of dioxane and 3.5 ml of c-HCl was added. The reaction mixture was refluxed for 4.5 h. The solvent was distilled off under reduced pressure. The residue product was washed with ether (3.7 g, 92.5%). To obtain the analytical sample the product was recrystallized from water. Recrystallization from DMSO afforded the colorless crystals suitable for X-ray diffraction. m.p. 176–178 °C; IR (KBr, cm^-1^) 3450 (NH), 3150 (NH), 3100, 3000, 2900 (CH), 1700 (C=O), 1610, 1500 (C=N); ^1^H NMR (DMSO-d_6_, p.p.m.) 11.3 (1*H*, b, NH), 6.4 (2*H*, b, NH_2_; ^13^C NMR (DMSO-d_6_, p.p.m.) 169.4 (C=N), 153.0 (C=O); Anal. Calcd. For C_2_H_3_N_3_OS: C 20.51, H 2.58, N 35.88, S 27.37. Found: C 20.19, H 2.65, N 34.28, S 27.22.

### Refinement

H atoms of the NH and NH_2_ groups were located in a difference Fourier map and refined freely [refined distances = 0.79 (2)–0.94 (2) Å].

### Crystal data

**Table:**

C_2_H_3_N_3_OS	*Z* = 6
*M**_r_* = 117.13	*F*(000) = 360
Triclinic, *P*1	*D*_x_ = 1.694 Mg m^−^^3^
Hall symbol: -P 1	Mo *K*α radiation, λ = 0.71073 Å
*a* = 7.2860 (2) Å	Cell parameters from 5437 reflections
*b* = 10.2982 (3) Å	θ = 2.2–26.1°
*c* = 10.7727 (3) Å	µ = 0.57 mm^−^^1^
α = 63.721 (3)°	*T* = 296 K
β = 73.122 (2)°	Block, colourless
γ = 76.737 (2)°	0.15 × 0.1 × 0.05 mm
*V* = 688.74 (3) Å^3^	

### Data collection

**Table:**

Bruker SMART CCD area-detector diffractometer	2526 reflections with *I* > 2σ(*I*)
Graphite monochromator	*R*_int_ = 0.055
φ and ω scans	θ_max_ = 28.3°, θ_min_ = 2.2°
Absorption correction: multi-scan (*SADABS*; Bruker, 2002)	*h* = −9→9
*T*_min_ = 0.93, *T*_max_ = 0.97	*k* = −13→13
23857 measured reflections	*l* = −14→14
3433 independent reflections	

### Refinement

**Table:**

Refinement on *F*^2^	Primary atom site location: structure-invariant direct methods
Least-squares matrix: full	Secondary atom site location: difference Fourier map
*R*[*F*^2^ > 2σ(*F*^2^)] = 0.031	Hydrogen site location: inferred from neighbouring sites
*wR*(*F*^2^) = 0.080	All H-atom parameters refined
*S* = 0.94	*w* = 1/[σ^2^(*F*_o_^2^) + (0.0423*P*)^2^] where *P* = (*F*_o_^2^ + 2*F*_c_^2^)/3
3433 reflections	(Δ/σ)_max_ < 0.001
226 parameters	Δρ_max_ = 0.30 e Å^−^^3^
0 restraints	Δρ_min_ = −0.26 e Å^−^^3^

### Special details

**Table:**

Geometry. All s.u.'s (except the s.u. in the dihedral angle between two l.s. planes) are estimated using the full covariance matrix. The cell s.u.'s are taken into account individually in the estimation of s.u.'s in distances, angles and torsion angles; correlations between s.u.'s in cell parameters are only used when they are defined by crystal symmetry. An approximate (isotropic) treatment of cell s.u.'s is used for estimating s.u.'s involving l.s. planes.
Refinement. Refinement of *F*^2^ against ALL reflections. The weighted *R*-factor *wR* and goodness of fit *S* are based on *F*^2^, conventional *R*-factors *R* are based on *F*, with *F* set to zero for negative *F*^2^. The threshold expression of *F*^2^ > 2σ(*F*^2^) is used only for calculating *R*-factors(gt) *etc*. and is not relevant to the choice of reflections for refinement. *R*-factors based on *F*^2^ are statistically about twice as large as those based on *F*, and *R*- factors based on ALL data will be even larger.

### Fractional atomic coordinates and isotropic or equivalent isotropic displacement parameters (Å^2^)

**Table:**

	*x*	*y*	*z*	*U*_iso_*/*U*_eq_	
S1	0.86165 (6)	−0.07773 (5)	−0.06572 (4)	0.03647 (13)	
C2	0.7587 (2)	0.10880 (18)	−0.13554 (16)	0.0339 (4)	
N3	0.6466 (2)	0.13649 (15)	−0.02585 (14)	0.0329 (3)	
H3	0.580 (3)	0.219 (2)	−0.0362 (19)	0.044 (5)*	
N4	0.62764 (19)	0.02538 (14)	0.10886 (13)	0.0303 (3)	
C5	0.7333 (2)	−0.09181 (17)	0.10313 (16)	0.0289 (3)	
O6	0.7853 (2)	0.19397 (15)	−0.26078 (12)	0.0517 (4)	
N7	0.7416 (3)	−0.21857 (17)	0.21825 (17)	0.0472 (4)	
H7A	0.825 (3)	−0.291 (3)	0.210 (2)	0.072 (7)*	
H7B	0.672 (3)	−0.224 (2)	0.297 (2)	0.052 (6)*	
S8	0.15828 (7)	0.02436 (5)	0.59226 (4)	0.03889 (13)	
C9	0.3260 (2)	−0.05097 (18)	0.47618 (16)	0.0338 (4)	
N10	0.3484 (2)	0.05559 (15)	0.34594 (14)	0.0353 (3)	
H10	0.439 (3)	0.047 (2)	0.262 (2)	0.065 (6)*	
N11	0.2481 (2)	0.19183 (14)	0.32756 (13)	0.0354 (3)	
C12	0.1437 (2)	0.19073 (18)	0.44702 (16)	0.0348 (4)	
O13	0.40594 (19)	−0.17651 (13)	0.51031 (13)	0.0485 (3)	
N14	0.0375 (3)	0.3114 (2)	0.4619 (2)	0.0592 (5)	
H14A	−0.048 (3)	0.297 (2)	0.536 (3)	0.070 (7)*	
H14B	0.019 (3)	0.387 (2)	0.386 (2)	0.050 (6)*	
S15	0.23799 (7)	0.66689 (5)	−0.15169 (5)	0.04302 (14)	
C16	0.1723 (2)	0.55543 (18)	0.03347 (17)	0.0363 (4)	
N17	0.2688 (2)	0.42407 (15)	0.05437 (15)	0.0353 (3)	
H17	0.262 (3)	0.352 (2)	0.138 (2)	0.053 (6)*	
N18	0.3933 (2)	0.40035 (14)	−0.06009 (13)	0.0354 (3)	
C19	0.3895 (2)	0.51734 (17)	−0.17336 (17)	0.0356 (4)	
O20	0.0594 (2)	0.59341 (14)	0.12439 (14)	0.0537 (4)	
N21	0.4930 (3)	0.5249 (2)	−0.30232 (18)	0.0651 (6)	
H21A	0.492 (4)	0.606 (3)	−0.363 (3)	0.081 (8)*	
H21B	0.566 (3)	0.455 (3)	−0.304 (2)	0.071 (8)*	

### Atomic displacement parameters (Å^2^)

**Table:**

	*U*^11^	*U*^22^	*U*^33^	*U*^12^	*U*^13^	*U*^23^
S1	0.0365 (2)	0.0374 (2)	0.0315 (2)	0.00386 (18)	0.00096 (17)	−0.01947 (18)
C2	0.0319 (9)	0.0371 (9)	0.0289 (8)	−0.0040 (7)	−0.0003 (7)	−0.0139 (7)
N3	0.0386 (8)	0.0250 (7)	0.0254 (6)	0.0032 (6)	−0.0008 (6)	−0.0089 (6)
N4	0.0345 (7)	0.0262 (7)	0.0233 (6)	0.0000 (6)	−0.0004 (5)	−0.0091 (5)
C5	0.0290 (8)	0.0292 (8)	0.0274 (7)	−0.0007 (7)	−0.0024 (6)	−0.0139 (7)
O6	0.0631 (9)	0.0485 (8)	0.0246 (6)	−0.0049 (7)	0.0027 (6)	−0.0064 (6)
N7	0.0625 (12)	0.0306 (9)	0.0324 (8)	0.0104 (8)	−0.0046 (8)	−0.0096 (7)
S8	0.0458 (3)	0.0368 (2)	0.02102 (19)	−0.00454 (19)	0.00086 (17)	−0.00531 (17)
C9	0.0375 (9)	0.0317 (9)	0.0270 (8)	−0.0028 (7)	−0.0059 (7)	−0.0084 (7)
N10	0.0412 (8)	0.0293 (7)	0.0241 (6)	0.0039 (6)	−0.0013 (6)	−0.0080 (6)
N11	0.0412 (8)	0.0276 (7)	0.0224 (6)	0.0029 (6)	0.0011 (6)	−0.0052 (6)
C12	0.0366 (9)	0.0310 (9)	0.0261 (8)	−0.0022 (7)	0.0009 (7)	−0.0078 (7)
O13	0.0601 (9)	0.0290 (7)	0.0423 (7)	0.0049 (6)	−0.0128 (6)	−0.0056 (6)
N14	0.0692 (13)	0.0386 (10)	0.0393 (10)	0.0088 (9)	0.0126 (9)	−0.0111 (8)
S15	0.0533 (3)	0.0232 (2)	0.0365 (2)	0.00734 (19)	−0.0052 (2)	−0.00628 (18)
C16	0.0386 (10)	0.0298 (9)	0.0342 (9)	0.0008 (7)	−0.0040 (7)	−0.0121 (7)
N17	0.0417 (9)	0.0258 (7)	0.0255 (7)	0.0029 (6)	−0.0009 (6)	−0.0061 (6)
N18	0.0423 (8)	0.0241 (7)	0.0267 (7)	0.0036 (6)	0.0006 (6)	−0.0074 (6)
C19	0.0428 (10)	0.0240 (8)	0.0305 (8)	0.0000 (7)	−0.0020 (7)	−0.0083 (7)
O20	0.0561 (9)	0.0453 (8)	0.0474 (7)	0.0072 (6)	0.0046 (6)	−0.0238 (6)
N21	0.0938 (16)	0.0328 (10)	0.0314 (9)	0.0067 (10)	0.0134 (9)	−0.0036 (8)

### Geometric parameters (Å, º)

**Table:**

S1—C5	1.7449 (15)	N10—H10	0.98 (2)
S1—C2	1.7905 (17)	N11—C12	1.2874 (19)
C2—O6	1.2270 (19)	C12—N14	1.354 (2)
C2—N3	1.333 (2)	N14—H14A	0.83 (2)
N3—N4	1.3857 (18)	N14—H14B	0.86 (2)
N3—H3	0.854 (19)	S15—C19	1.7419 (17)
N4—C5	1.2905 (19)	S15—C16	1.7876 (17)
C5—N7	1.349 (2)	C16—O20	1.2298 (19)
N7—H7A	0.87 (2)	C16—N17	1.327 (2)
N7—H7B	0.84 (2)	N17—N18	1.3853 (18)
S8—C12	1.7419 (16)	N17—H17	0.88 (2)
S8—C9	1.7874 (17)	N18—C19	1.2821 (19)
C9—O13	1.2264 (19)	C19—N21	1.352 (2)
C9—N10	1.336 (2)	N21—H21A	0.80 (3)
N10—N11	1.3817 (18)	N21—H21B	0.79 (2)
			
C5—S1—C2	88.70 (7)	C12—N11—N10	110.16 (13)
O6—C2—N3	126.79 (16)	N11—C12—N14	123.00 (15)
O6—C2—S1	126.30 (13)	N11—C12—S8	115.37 (12)
N3—C2—S1	106.90 (12)	N14—C12—S8	121.53 (13)
C2—N3—N4	119.16 (14)	C12—N14—H14A	116.1 (16)
C2—N3—H3	122.2 (13)	C12—N14—H14B	117.8 (13)
N4—N3—H3	118.5 (13)	H14A—N14—H14B	118 (2)
C5—N4—N3	109.74 (12)	C19—S15—C16	88.49 (8)
N4—C5—N7	122.96 (15)	O20—C16—N17	126.47 (16)
N4—C5—S1	115.48 (12)	O20—C16—S15	126.48 (13)
N7—C5—S1	121.54 (12)	N17—C16—S15	107.05 (12)
C5—N7—H7A	118.8 (15)	C16—N17—N18	119.02 (14)
C5—N7—H7B	119.4 (14)	C16—N17—H17	122.9 (13)
H7A—N7—H7B	122 (2)	N18—N17—H17	118.0 (13)
C12—S8—C9	88.73 (7)	C19—N18—N17	109.75 (13)
O13—C9—N10	126.70 (16)	N18—C19—N21	122.74 (16)
O13—C9—S8	126.29 (13)	N18—C19—S15	115.68 (12)
N10—C9—S8	107.01 (12)	N21—C19—S15	121.57 (13)
C9—N10—N11	118.72 (13)	C19—N21—H21A	113.9 (17)
C9—N10—H10	125.0 (12)	C19—N21—H21B	116.2 (17)
N11—N10—H10	116.1 (12)	H21A—N21—H21B	128 (2)

### Hydrogen-bond geometry (Å, º)

**Table:**

*D*—H···*A*	*D*—H	H···*A*	*D*···*A*	*D*—H···*A*
N3—H3···N18	0.854 (19)	2.004 (19)	2.8516 (19)	171.9 (18)
N7—H7*A*···O20^i^	0.87 (2)	2.07 (2)	2.907 (2)	160 (2)
N10—H10···N4	0.98 (2)	1.88 (2)	2.8558 (19)	175.7 (18)
N14—H14*A*···O6^ii^	0.83 (2)	2.10 (2)	2.897 (2)	162 (2)
N17—H17···N11	0.88 (2)	1.97 (2)	2.8424 (18)	179 (4)
N21—H21*A*···O13^iii^	0.80 (3)	2.10 (3)	2.878 (2)	163 (2)

Symmetry codes: (i) *x*+1, *y*−1, *z*; (ii) *x*−1, *y*, *z*+1; (iii) *x*, *y*+1, *z*−1.
